# EQ-5D-3L Derived Population Norms for Health Related Quality of Life in Sri Lanka

**DOI:** 10.1371/journal.pone.0108434

**Published:** 2014-11-03

**Authors:** Sanjeewa Kularatna, Jennifer A. Whitty, Newell W. Johnson, Ruwan Jayasinghe, Paul A. Scuffham

**Affiliations:** 1 Centre for Applied Health Economics, School of Medicine, Griffith University, Brisbane, Queensland, Australia; 2 Population and Social Health Research Programme, Griffith Health Institute, Griffith University, Brisbane, Queensland, Australia; 3 School of Pharmacy, The University of Queensland, Brisbane, Queensland, Australia; 4 Faculty of Dental sciences, University of Peradeniya, Peradeniya, Sri Lanka; University of Bremen, Germany

## Abstract

**Background:**

Health Related Quality of Life (HRQoL) is an important outcome measure in health economic evaluation that guides health resource allocations. Population norms for HRQoL are an essential ingredient in health economics and in the evaluation of population health. The aim of this study was to produce EQ-5D-3L-derived population norms for Sri Lanka.

**Method:**

A population sample (n =  780) was selected from four districts of Sri Lanka. A stratified cluster sampling approach with probability proportionate to size was employed. Twenty six clusters of 30 participants each were selected; each participant completed the EQ-5D-3L in a face-to-face interview. Utility weights for their EQ-5D-3L health states were assigned using the Sri Lankan EQ-5D-3L algorithm. The population norms are reported by age and socio-economic variables.

**Results:**

The EQ-5D-3L was completed by 736 people, representing a 94% response rate. Sixty per cent of the sample reported being in full health. The percentage of people responding to any problems in the five EQ-5D-3L dimensions increased with age. The mean EQ-5D-3L weight was 0.85 (SD 0.008; 95%CI 0.84-0.87). The mean EQ-5D-3L weight was significantly associated with age, housing type, disease experience and religiosity. People above 70 years of age were 7.5 times more likely to report mobility problems and 3.7 times more likely to report pain/discomfort than those aged 18-29 years. Those with a tertiary education were five times less likely to report any HRQoL problems than those without a tertiary education. A person living in a shanty was 4.3 more likely to have problems in usual activities than a person living in a single house.

**Conclusion:**

The population norms in Sri Lanka vary with socio-demographic characteristics. The socioeconomically disadvantaged have a lower HRQoL. The trends of population norms observed in this lower middle income country were generally similar to those previously reported in high income countries.

## Introduction

Measures of Health-related Quality of Life (HRQoL) are increasingly recognised as important in the provision of measurable outcomes for health interventions. They are an essential component of evidence-based public health policy, aspiring to the ultimate goal of health for all [1]. Having a national HRQoL baseline measure provides planners with a common benchmark for assessing improvements in public health, and can provide an overall indicator of quality of care [2]. Analysis of HRQoL data helps to identify needs for new or revised health policies, for the allocation of health resources, guides strategic planning and helps to improve the monitoring of the outcome of community health interventions. Therefore, HRQoL has evolved into a valid indicator of service needs and intervention outcomes and is an established component of health surveillance in many countries[3]. HRQoL includes the physical and mental well-being of people. Physical and mental health is affected by socioeconomic status, health care policies, risk behaviours and social support systems [4]. The outcome of health interventions can be measured by the extent of the changes in a HRQoL instrument. Routine collection of HRQoL data is important for all aspects of health care decision making.

The EQ-5D-3L is the most popular generic preference-based instrument to measure utility and has the most number of country specific valuations reported around the world [5]. The EQ-5D-3L describes HRQoL in each of five dimensions; mobility, self-care, usual activities, pain/discomfort and anxiety/depression. Each dimension is described by a single item which is divided into three levels; no problem, moderate problems and severe problems [6]. Combinations of the five dimensions and three levels produce 243 health states. In health state valuations, a utility weight for each health state is estimated. Utility weights are the building block of Quality Adjusted Life Years (QALYs), a cardinal measure of health outcome combining both survival and HRQoL. Utility weights denote a preference for a health state on a 0-1 scale where 0 is death and 1 is full health. Thus the EQ-5D-3L health state for full health is 11111, and the worst is 33333. In addition, health states which are considered worse than death have values from 0 to -1.

The UK [2], [7], USA [8], [9], Sweden [10], Denmark [11], and Australia [12], [13], among other high income countries, have reported EQ-5D-3L utility valuations and descriptions of population norms derived therefrom, as have China [14] and Singapore [15]. Population norms allow a comparison of the HRQoL of patients with that of an average person in the community [15], assess the incremental effect of interventions when a control group is not available [12], provide an index value for normal health for a specific socio-demographic group of people [12] and support a comparison of population subgroups to explore equity concerns [16]. Moreover, they can be used to estimate the utility detriment in acute onset conditions. Population norms are an essential baseline for estimating outcomes in evaluation of health programmes and economic evaluations of health interventions.

Sri Lanka, an island nation, had a mean income per capita in 2012 of US$6046, with 3.4% of its GDP spent on health [17]. Sinhalese (75%) are the major ethnic group, with Tamils and Muslims forming the rest of the population [18]. Life expectancy at birth is 71 years for males and 78 years for females[17]. Although it is a lower middle income country, its health indicators are considered to be amongst the best in the South Asian region, with a maternal mortality rate of 37.7: 100,000 live births[19], and 99% immunisation coverage [20]. In the last three decades Sri Lanka went through a devastating civil war. However, with peace and a strengthening economy there are better prospects of life for most people. A longer life expectancy, however, means more people are living with morbidity [14]. This will increase chronic, non-communicable diseases (NCD) such as cancer, cardiovascular diseases and mental health problems associated with changing social values.

In recent times Sri Lanka has reported HRQoL studies on liver disease [21], spinal cord injuries [22], parasitic diseases [23], oral health [24], vision [25] and cancer [26]. These studies have provided better grounds for clinicians to understand the quality of life issues associated with their patients. Recent studies in Sri Lanka have generated valuations for the EQ-5D-3L health states and EORTC-8D health states, providing a better framework to use HRQoL in policy decisions [27].The major national surveys conducted in Sri Lanka are the 10 yearly Census of Population and Housing (most recent in 2012) and the Demographic and Health Survey carried out by the National Statistics and Survey Department [28]. Unfortunately, these surveys do not collect HRQoL data. We assert that it is time Sri Lanka also moved from mortality-based health indicators to morbidity-based health indicators, given its improving life expectancy and higher NCD burden [14]. Thus, the measurement of health status in Sri Lanka is a pressing need.

At the moment, population norms for HRQoL in Sri Lanka do not exist. The observed difference of EQ-5D-3L utility weights between low and high income countries [29] pose the idea that the Sri Lankan EQ-5D-3L population norms would also be different from the high income countries. Therefore, publication of EQ-5D-3L population norms would be advantageous for Sri Lankan decision makers and researchers. The aim of this study is to estimate EQ-5D-3L derived population norms for Sri Lanka using a large population sample. This will also facilitate an international comparison of HRQoL in Sri Lanka with other preference-based population norms within the world context.

## Methods

The data were collected alongside an EQ-5D-3L health state valuation study in Sri Lanka, from a population sample of 780 persons, drawn from four districts, selected purposively to support diversity, as logistic and financial constraints prevented data collection from all districts [27]. The districts chosen were Colombo (the most populated and metropolitan district), Kandy (a predominantly urban population representing the central part of the country), Kalutara (a mix of suburban and rural areas) and Kurunegala (a rural district from the north western area of the island). The total population of these four districts were 5.2million according to the 2011 census [30]. Eighty two percent Sinhalese, 7% Tamils and 9% Muslims lived in them compared to national values of 82% Sinhalese, 9% Tamils and 8% Muslims[30]. The four districts together contained 32% of the total population of the country [18]. Ethical approval was granted from the ethical committee of the Sri Lanka Medical Association (ERC/12/022) and Griffith University Human Research Ethics Committee (MED/29/12/HREC). The participants provided written informed consent before the commencement of data collection. The participants signed the first sheet of the data collection instrument giving their consent. The participants were given a copy of the information sheet. The ethics committees approved the consent procedure.

Detailed methods have been reported elsewhere [27]. In short, stratified cluster sampling with probability proportionate to size was used to select the sample [27]. Twenty six clusters of 30 each were used. A cluster consisted of a public health midwife area (PHM): the smallest area in the Sri Lankan health administrative system. The sample of 780 was proportionately allocated to four districts, according to population size. To give each household an equal probability of selection, cluster sampling with probability proportionate to size (PPS) was carried out to select the sample within the district. A cumulative list of PHM areas was created. After a random start, PHM areas were selected systematically. Thirty households were selected randomly from each PHM area using a voters list. One respondent from a household was selected using the Kish grid method [27]. If the occupants were absent in a selected household, data collectors made repeated visits during the time they were in the area. If the occupants were not contactable during this time period they were considered non-respondents and no replacements were made. Eight trained associate investigators collected information on demography, family income, morbidity and religiosity. The latter was measured by the Duke University Religion Index (DUREL) instrument [31], our hypothesis being that this, in a multi faith society, would influence self-reported HRQoL of the Sri Lankan population.

The data were collected over 2 months in 2012-2013. Face-to-face interviews were carried out in the participants' household either in Sinhalese or English language. The respondents answered a structured questionnaire which captured socio-demographic characteristics. Then they were asked to rate items within the EQ-5D-3L questionnaire for their current health state. The EQ-5D-3L questionnaire was validated for Sri Lanka to be used in Sinhalese language [5]. In addition, they responded to the EQ-5D-3L visual analogue scale (VAS), a thermometer like indicator to record current health [32]. This scale ranged from 0 (worst imaginable health state) – 100 (the best imaginable health state).The participants were requested to point out “which point best fits your own health state today”.

### Data analysis

Analysis was carried out using Stata 12.0. The data were analysed unweighted. The EUROQoL group states “as the population norms are represented by age and gender there is no need for the sample to have the same age distribution as the general population” [6]. The frequency of people reporting no problems, moderate problems and severe problems for each dimension were calculated and the percentage of people reporting any problem in each dimension was calculated for the total sample and stratified by various demographic variables. Chi square tests were used to determine the significance between groups in categorical variables.

The self-reported EQ-5D-3L health state utility weight for each respondent was calculated using Sri Lankan EQ-5D-3L values in a forthcoming publication. Mean EQ-5D-3L weights for the sample and categorical demographic variables were summarised and ANOVA was used for comparisons in the analysis of these profile data. Logistic regression was then used to investigate the association between having any problem in each dimension and socio-demographic variables. Using a stepwise function all independent socio-demographic variables were tested in the logistic regression model. Only the variables with p<0.1 were retained and their sub categories examined. The significant variables were considered as the main effects and subsequent interactions among them were tested in a logistic regression model. The results are presented as odds ratios (OR).

In the analysis, the sample was divided into six age groups (18–29 years, 30–39, 40–49 etc.) to aid comparison with published population norms from other countries. Though data were collected for six educational categories, they were divided into four: no formal education; primary education (up to grade 8); secondary education (completion of either grade 10 or 12), tertiary (any diploma or degree). Marital status was also summarised to three categories from six; never married, married (including living together), widowed/separated. The three major ethnicities of Sinhalese, Tamils and Muslims were considered for the analysis with an “other” category for minor ethnicities. Dummy variables were constructed for any problems in each dimension of the EQ-5D-3L representing level 2 or level 3. A dummy variable was also used for any current disease to include all people who self-reported suffering from any NCD. The religiosity questions were converted according to the scoring instructions for the DUREL [31]. The answers of the first two questions which asked the frequency of religious activities in public and in private respectively were reversed. Answers to the second section, which examined intensity of religious beliefs, were reversed, added together and the total score of the three sub-sections used for analysis.

## Results

From the sample of 780, there were 736 responses to the EQ-5D-3L questionnaire (94% response rate). Of the 736 responses, there were 719 (92%) with complete data, of which 63% were female (Table 1). The sample distribution among the age groups was equal. The majority were Sinhalese (91%). Five percent of the sample did not have any formal education. Only 4% of the sample lived in huts or shanties. Only 37% of the sample was employed. Of the 719, 39% of the participants reported suffering from a chronic NCD and accidents/injury. Of the sample 17% had hypertension, 19% had diabetes and four people reported as having cancer. Only 52% had not visited a doctor for the last 30 days for some form of treatment. Twenty percent of the sample had been admitted to a hospital for treatment over the last year. There were 13 participants who were admitted to a hospital three or more than three times over that time period (Table 1). The sample had more females (62.5%) than national values (51%).

**Table 1:** Socio-demographic characteristics of the sample n = 719.

Variable	n (%)	Sri Lankan population*
Sex		
Male	269 (37.41)	51.5
Female	450 (62.59)	48.5
Age		
18–29	128(17.8)	
30–39	128(17.8)	
40–49	132(18.36)	
50–59	135(18.78)	
60–69	114(15.86)	
70+	82 (11.4)	
Ethnicity		
Sinhala	656(91.24)	74.9
Tamil	14 (1.95)	15.4
Muslim	47 (6.54)	9.2
Education		
No formal education	37 (5.18)	
Primary	157(22)	
Secondary	455 (63.7)	
Tertiary	65 (9.1)	
Housing type		
Single house	605(84.73)	
Flat/apartment	80(11.2)	
Hut/shanty	29(4.1)	
Annual household income		
0–99,999	123(17.42)	
100,000–199,999	169(23.94)	
200,00–299,999	124(17.56)	
300,000–399,999	105(14.87)	
Above 400,000	78(11.04)	
Preferred not to answer	107(15.16)	
Employment		
employed	254 (37.3)	
Non-economic activities	402 (59)	
Family worker	24(3.5)	
Marital status		
Never married	101(14)	
Married	595(82.8)	
Widow/divorced	23(3.2)	
District		
Colombo	209(29.1)	
Kandy	167(23.2)	
Kurunegala	196(27.3)	
Kalutara	147(20.45)	
Religion		
Buddhist	637(88.6)	70.2
Hindu	7(1)	12.6
Islam	55(7.6)	9.7
Christian	20(2.8)	6.1
Current disease n = 719		
Hypertension	125(17.4)	
Diabetes	134(18.6)	
Asthma	34(4.7)	
Epilepsy	2(0.3)	
Anaemia	3(0.4)	
Renal condition	9(1.3)	
Cardiac condition	27(3.8)	
Accident/Injury	6(0.8)	
Mental health	8(1.1)	
Gastro Intestinal problems	16(2.2)	
Skin disease	7(1)	
Cancer	4(0.6)	
Other	110(15.3)	
Number of visits to the doctor in the last month n = 697		
0	361(51.79)	
1	249(35.72)	
2	55(7.9)	
3	19(2.73)	
4 and above	13(1.87)	
Number of hospital admissions in the last year n = 703		
0	561(79.8)	
1	107(15.22)	
2	22(3.13)	
3 and above	13(1.85)	

* provisional 2012 census available results [18]; *15–59 age groups percentage was 62%; above 60 years was 12.2%.

**Figure 1:**
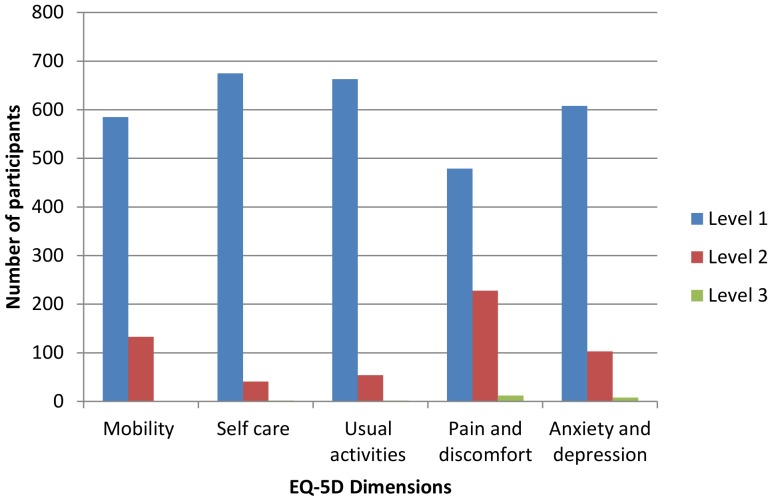
Health of participants described with the EQ-5D-3L instrument.

Figure 1 shows the reported problems facing the participants in the five dimensions of the EQ-5D-3L. The majority of participants did not report any problems (level 1). Only a small percentage had severe problems (level 3): 0.14% in mobility; 0.28% in self-care; 0.28% in usual care; 1.67% in pain/discomfort; 1.11% in anxiety/depression. The largest number of any problems was reported for pain and discomfort while the smallest number of any problems was reported for anxiety or depression. The percentage of people reporting themselves to be in full health was 60.5%.

The percentage of participants complaining of “any problems” (level 2 or 3) increased with age in all five dimensions (Table 2). The association with age and reporting any problem in each dimension was significant (p<0.0001). There was no perceptible difference between males and females having any problem in all dimensions except for pain and discomfort, where females had a higher number of complaints. However, the observed difference was not significant.

**Table 2:** Frequency and percentage of respondents reporting any problem on the 5 dimensions by age and gender.

	Total	p Value^1^	sex	
			Male	Female
Mobility				
Total	134(18.64)		45(16.7)	89(19.8)
Age group		0.000		
18–29	3(2.34)		1(0.78)	2(1.56)
30–39	14(10.94)		2(1.56)	12(9.38)
40–49	19(14.39)		1(0.76)	18(13.64)
50–59	30(22.22)		11(8.15)	19(14.07)
60–69	35(30.7)		14(12.28)	21(18.42)
70+	33(40.24)		16(19.51)	17(20.73)
Self-care		0.000		
Total	43(5.99)		17(6.34)	26(5.78)
Age group				
18–29	1(0.78)		1(0.78)	0
30–39	1(0.78)		0	1(0.78)
40–49	5(3.79)		1(0.75)	4(3.03)
50–59	8(5.93)		4(2.96)	4(2.96)
60–69	12(10.62)		5(4.43)	7(6.2)
70+	16(19.51)		6(7.32)	10(12.2)
Usual activities		0.000		
Total	56(7.79)		24(8.92)	32(7.11)
Age group				
18–29	0		0	0
30–39	2(1.56)		1(0.78)	1(0.78)
40–49	11(8.33)		2(1.52)	9(6.82)
50–59	8(5.93)		5(3.70	3(2.22)
60–69	13(11.4)		6(5.26)	7(6.14)
70+	22(26.88)		10(12.2)	12(14.63)
Pain and discomfort		0.000		
Total	240(33.38)		80(29.74)	160(35.56)
Age group				
18–29	11(8.59)		2(1.56)	9(7.03)
30–39	24(18.75)		3(2.34)	21(16.41)
40–49	46(34.85)		11(8.33)	35(26.52)
50–59	60(44.44)		20(14.82)	40(29.63)
60–69	55948.25)		24(21.05)	31(27.19)
70+	44(53.66)		20(24.39)	24(29.27)
Anxiety & depression		0.000		
Total	111(15.44)		39(14.5)	72(16.00)
Age group				
18–29	9(7.03)		1(0.78)	8(6.25)
30–39	13(10.16)		3(2.34)	10(7.81)
40–49	18(13.64)		7(5.3)	11(8.33)
50–59	30(22.22)		11(8.15)	19(14.07)
60–69	19(16.67)		9(7.9)	10(8.77)
70+	22(26.83)		8(9.76)	14(17.07)

1 Chi Square test; tested difference between categories in each socio-economic variable p<0.05 indicate significance.

The mean EQ-5D-3L score for the sample was 0.85 (SD 0.008, 0.84–0.87 95% CI). The mean EQ-5D-3L VAS score for the sample was 0.81 (SD 0.01, 0.79–0.85 95% CI). The means of the EQ-5D-3L weight differed significantly (p<0.0001) among the different levels of demographic variables given in Table 3, with the exceptions of annual household income, employment, ethnicity, district and religion. Between age groups, the mean EQ-5D-3L scores differed significantly (p<0.0001). There was significant difference in mean EQ-5D-3L scores between education groups (p<0.0001). In addition, the mean scores had a significant difference between the type of house lived in (p<0.0001). Experience with health care and morbidity groups also reported significant mean difference from each other (p<0.0001 People with any current NCD had a significantly different EQ-5D-3L score than others (p<0.0001). People who indulge in moderate amounts of religious activities reported the different mean scores from others HRQoL (p<0.016). There was significantly different utility scores from people with a renal condition (0.50) and those with mental health problems (0.53) (Table 3) compared with others.

**Table 3:** The EQ-5D-3L weight by demographic variables.

	Total Mean (SD)	P value 1	Male	Female
Total	0.85(0.21)		0.86(0.23)	0.85(0.20)
Age group		0.000		
18–29	0.95(0.15)		0.96(0.20)	0.95(0.10)
30–39	0.91(0.16)		0.94(0.18)	0.91(0.15)
40–49	0.87(0.17)		0.91(0.12)	0.85(0.19)
50–59	0.82(0.21)		0.82(0.22)	0.83(0.2)
60–69	0.8(0.25)		0.82(0.27)	0.78(0.23)
70+	0.73(0.27)		0.77(0.27)	0.70(0.28)
Ethnicity		0.770		
Sinhala	0.85(0.21)		0.86(0.24)	0.86(0.2)
Tamil	0.91(0.13)		0.93(0.11)	0.87(0.16)
Muslim	0.84(0.19)		0.91(0.13)	0.79(0.21)
other	0.89(0.14)		0.79	
Education		0.000		
No formal education	0.7(0.28)		0.68(0.31)	0.72(0.27)
Primary	0.78(0.25)		0.79(0.29)	0.78(0.23)
Secondary	0.89(0.18)		0.91(0.19)	0.88(0.17)
Tertiary	0.93(0.12)		0.93(0.12)	0.92(0.12)
Housing type		0.000		
Single house	0.86(0.21)		0.87(0.22)	0.86(0.19)
Flat/Apartment	0.91(0.14)		0.92(0.13)	0.9(0.14)
Hut/Shanty	0.66(0.33)		0.65(0.38)	0.67(0.27)
Annual household income		0.161		
0–99,999	0.85(0.21)		0.83(0.24)	0.86(0.19)
100,000–199,999	0.87(0.2)		0.89(0.19)	0.85(0.2)
200,000–299,999	0.87(0.23)		0.86(0.29)	0.87(0.19)
300,000–399,999	0.89(0.16)		0.92(0.15)	0.87(0.17)
400,000 and above	0.86(0.21)		0.88(0.21)	0.84(0.2)
Prefer not to answer	0.81(0.25)		0.81(0.27)	0.81(0.24)
Employment		0.100		
Employed	0.89(0.18)		0.9(0.18)	0.86(0.18)
Non-economic activities	0.85(0.23)		0.81(0.29)	0.86(0.21)
Family worker	0.87(0.17)		0.89(0.14)	0.84(0.19)
Marital Status				
Never Married	0.93(0.19)		0.92(0.26)	0.95(0.11)
Married	0.85(0.21)		0.85(0.22)	0.84(0.2)
Widow/divorced	0.78(0.24)		0.83(0.3)	0.77(0.22)
District		0.240		
Colombo	0.87(0.19)		0.88(0.23)	0.86(0.67)
Kandy	0.87(0.19)		0.89(0.19)	0.86(0.19)
Kurunegala	0.83(0.24)		0.81(0.27)	0.85(0.23)
Kalutara	0.86(0.2)		0.87(0.20)	0.85(0.2)
Religion		0.340		
Buddhist	0.85(0.22)		0.86(0.24)	0.85(0.2)
Hindu	0.93(0.11)		0.95(0.11)	0.89(0.15)
Islam	0.86(0.18)		0.92(0.12)	0.82(0.21)
Christian	0.93(0.13)		0.96(0.08)	0.92(0.14)
Any Disease experience		0.000		
No disease experience	0.92(0.14)		0.94(0.12)	0.91(0.15)
Any disease experience	0.75(0.25)		0.72(0.3)	0.76(0.23)
Disease				
Hypertension	0.73(0.27)			
Diabetes	0.72(0.09)			
Asthma	0.79(0.20)			
Renal condition	0.50(0.21)			
Cardiac condition	0.64(0.23)			
Accident/Injury	0.59(0.30)			
Mental health	0.53(0.35)			
Gastro Intestinal problems	0.76(0.24)			
Skin disease	0.94(0.14)			
Doctor visits within the last 30 days		0.000		
0	0.91(0.16)		0.92(0.16)	0.89(0.16)
1	0.82(0.22)		0.81(0.24)	0.82(0.21)
2	0.78(0.28)		0.74(0.38)	0.8(0.23)
3	0.67(0.26)		0.73(0.23)	0.64(0.28)
4 or more	0.67(0.41)		0.42(0.56)	0.78(0.31)
Hospital visits		0.000		
0	0.88(0.18)		0.89(0.2)	0.88(0.17)
1	0.79(0.25)		0.8(0.31)	0.79(0.23)
2	0.67(0.3)		0.75(0.21)	0.61(0.36)
3 and above	0.58(0.32)			0.66(0.27)
Frequency of public religious activities		0.016		
Never	0.81(0.37)		0.77(0.46)	0.89(0.15)
Once a year or less	0.77(0.33)		0.7(0.38)	0.8(0.29)
A few times a year	0.84(0.22)		0.84(0.25)	0.84(0.18)
A few times a month	0.88(0.2)		0.89(0.21)	0.87(0.19)
Once a week	0.86(0.2)		0.9(0.18)	0.84(0.21)
More than once a week	0.82(0.19)		0.83(0.19)	0.81(0.2)
Frequency of private religious activities		0.006		
Rarely or never	0.91(0.16)		0.92(0.17)	0.9(0.12)
A few times a month	0.97(0.1)		1(0)	0.89(0.21)
Once a week	0.93(0.16)		1(0)	0.84(0.23)
Two or more times a week	0.87(0.19)		0.93(0.18)	0.83(0.18)
Daily	0.86(0.21)		0.85(0.24)	0.87(0.19)
More than once a day	0.81(0.22)		0.81(0.24)	0.81(0.21)

1 ANOVA; tested difference between categories in each socio-economic variable p<0.05 indicate significance.

The logistic regression provided Odds Ratios (OR) for the relationship between any problems reported for each dimension and socio-demographic variables (Table 4). There were no significant meaningful ORs for the interaction between main effects (p>0.1). In all five dimensions, gender, household income, and employment did not significantly affect the odds ratios of reporting any problems (p>0.1). Only the variables of any current morbidity, visits to GP within 30 days, and admission to hospital within the last year exhibited a significant association with reporting any problems in all five dimensions (p<0.05). Age had a significant association with all dimensions except anxiety and depression (p<0.05). People who are above 70 years old are 7.5 (2–2.8, 95% CI) times more likely to report mobility problems and 3.7 (95% CI 1.5–9.3,) times more likely to report pain and discomfort than people age 18– 29 (p<0.01). The likelihood of reporting mobility or pain and discomfort in those with a tertiary education are 5 (OR 0.2, 95% CI 0.04–0.9, p<0.01) and 3 (OR 0.3, 95% CI 0.1–0.9, p<0.05) times less likely respectively compared with people who had no formal education. The district people live in had a significant association with self-care and usual activities (p<0.05).The likelihood of reporting a problem of self-care was 5 (95% CI 1.4–17,) times higher for people living in Kurunegala than people living in Colombo (p<0.01). A person living in a hut or a shanty was 4.3 (95% CI 1.1–16,) times more likely to report a problem in usual activities than a person living in a single house (p<0.01).

**Table 4:** Logistic regression for any problems in the five dimensions with demographic variables.

	Mobility (OR)	Self-care	Usual activities	Pain and discomfort	Anxiety and depression
	OR (95% CI)	OR (95% CI)	OR (95% CI)	OR (95% CI)	OR (95% CI)
Age group					
18–29	Ref	Ref	Ref	Ref	
30–39	3.7(0.99–13)	0.6(.03–1.)	0.08(0.01–.53)**	1.1(.6–3.6)	
40–49	3.3(0.9–12)	1.8(0.17–19)	.24(0.07–.86)**	3.2(1.4–7.4)**	
50–59	4.4(1.2–16)**	1.5(0.14–15)	.16(0.05–.51)**	3.5(1.5–8.2)**	
60–69	5.3(1.5–20)**	3.1(0.31–29)	.25(0.09–.75)**	3.3(1.4–8)**	
70+	7.5(2–28)**	7.8(0.9–75)	1	3.7(1.5–9.3)**	
Ethnicity					
Sinhala				Ref	
Tamil				1.1(.12–10)	
Muslim				6.4(.9–43)	
other				3.1(.05–199	
Education					
No formal education	Ref	Ref	Ref	Ref	
Primary	1.2(0.5–3)	1.5(0.5–5.4)	2.4(0.6–10)	0.9(0.4–2.2)	
Secondary	0.6(0.2–1.4)	0.3(0.1–1.2)	0.9(.2–3.6)	0.5(0.2–1.1)	
Tertiary	0.2(0.04–0.9)**	0.3(0.02–3.8)	1	0.3(0.1–0.9)*	
Marital status					
Never Married				Ref	Ref
Married				1.7(.8–3.7)	1.0(0.5–2.2)
Widow/divorced				1.5(.4–5.3)	4.2(1.3–13.5)**
Religion					
Buddhist	Ref			Ref	
Hindu	–			0.5(0.04–7)	
Islam	1.2(.6–2.8)			0.17(0.03–1)	
Christian				0.18((.03–.9)**	
Any disease experience					
No	Ref	Ref	Ref	Ref	Ref
Yes	3.4(2–6)**	4.9(1.7–14)**	4(1.4–11)**	3.3(2.2–5)**	3(1.7–5)**
Visits to a doctor within last 30 days					
No	Ref		Ref		Ref
1	1.5(0.9–3)		2.1(.8–5.9)		1.4(.8–2.3)
2	2(0.9–5)		3.4(.9–12)		1.4(.6–3.3)
3	1.7(.5–6)		7.4(1.4–38)		7(2.3–20)**
4	1.9(.5–8)		2.6(.2–26)		1.8(.4–7.7)
Hospital within last year					
No	Ref	Ref	Ref	Ref	Ref
1	1.7(0.9–3.2)	3.8(1.5–9.4)**	3.3(1.3–8)**	2(1.2–3.6)**	.98(.5–1.9)
2	2.5(.86–7)	4.3(0.9–21)*	1.6(.3–8)	1.9(.7–5)	1.7(.57–4.9)
3 or more	4.2(1.1–16)**	3.2(0.6–17)	4.8(0.9–25)	3.4(0.9–14)*	4.5(1.3–16)**
District					
Colombo		Ref	Ref		
Kandy		.5(0.1–2.7)	.85(.2–3.5)		
Kurunegala		5(1.4–17)**	2.8(.8–9.4)*		
Kalutara		3.3(0.8–13)	2.4(.7–8.6)		
Frequency of public religious activities					
Never	Ref				
Once a year or less	1.5(.12–20)				
A few times a year	1.8(.16–21)				
A few times a month	1.3(1.6–14)				
Once a week	1.4(.12–16)				
More than once a week	3.4(.3–39)				
Frequency of private religious activities					
Rarely or never				Ref	
A few times a month				0.7(0.9–7)	
Once a week				0.4(0.3–5)	
Two or more times a week				2(.5–8)	
Daily				1.9(0.8–5)	
More than once a day				3.6(1.4–10)**	
House structure					
Single house		Ref	Ref		Ref
Flat/apartment		1	0.17(0.01–1.9)		0.86(.4–1.8)
Hut/Shanty		2.3 (.6–8.3)	4.3(1.1–16)**		3.1(1.2–8)**
Tenure					
Owned			Ref	Ref	Ref
Owned with mortgage			1	2.1(.4–11)	0.3(0.02–4.2)
Rented			23(3–173)**	2.9(.9–10)	5(1.6–16)**
Other			1.1(0.06–20)	1.6(0.4–7)	1.2(.2–8)
Internet access					
Yes			Ref		
No			0.4(.07–1.9)		

OR =  Odds Ratio; 95% CI =  95% confidence Interval; * – significant at p<0.05, ** – significant at p<0.01.

## Discussion

This study provides health status of a population sample from Sri Lanka using the EQ-5D-3L instrument. The values will therefore be useful as population norms to support the evaluation of health care in Sri Lanka. These values could be of importance to decision makers and outcome researchers in determining cost efficient health resource allocation. There are no other reported population norms to be compared with the present study.

The percentage of people who reported full health is 60.5% in the present study. This is similar to the observations from Konig et al. who reported 65% of a European (six countries) population sample did not indicate any problems in the EQ-5D-3L dimensions [33]. The EQ-5D-3L full health profile was less than 51% for a population sample from Sweden [10] and 58% in the UK [2]. However, the mean utility weight (0.85) and the mean VAS value (0.81) of the Sri Lankan population differ substantially from the Singapore mean utility weight (0.95) [15].The Sri Lankan mean utility weight is, however, similar to mean values of the UK (VAS) 82.5 [2]; The USA 0.87 [9]; Denmark 0.88 [11] and Sweden 0.85 [10]. These studies from high income countries were estimated from general population samples. Therefore, it can be reasonably suggested that the Sri Lankan population is on par with the general health status reported in the literature for high income countries. However, reported population norms could depend on the expectations of the society: of overall health is relatively poor in a country, then people's expectations are lower as they would be more stoic. This could be the case for much of South Asia. On the other hand in Western Europe, people's expectations and demands are at a higher level and will be more likely to complain. Moreover, Singapore, though Asian enjoys excellent healthcare with higher per capita income. However, being a very disciplined society are unlikely to complain.

In Sri Lanka the reported HRQoL declined with age. This is similar to observations made in China [14] Singapore [15] and other countries [2], [10]. The lack of a difference in observed health status between males and females in Sri Lanka is similar to Singapore [15] and Australia [34]. Our results contradict the popular belief that South Asian females are disadvantaged, at least on HRQoL grounds. On the other hand women in a South Asian country being long used to treated as second class citizens are less likely to complain about their HRQoL. However, this is different to a study in Denmark [11] in which men reported higher HRQoL than women in all age groups. In contrast, in Sweden [10] there was no significant difference for HRQOL between genders. People who never married recorded the highest HRQoL in our study. This observation was on par with the UK study [2] (which recorded more problems with widowed/separated/divorced) as well as the Singaporean [15] study.

Household income was not a significant factor in the present analysis. However, the self-reported household income data should be considered with caution as people could have given underestimates to a stranger asking about their wealth. The substantial wealth people generate from their non-formal economic activities in rural regions might not have been captured in our study. However, in high income countries, household income is a good indicator of health status [9]. People with a lower income in the UK [2], Sweden [10], Singapore [15] and USA [9] reported lower health status than people with higher incomes. A reasonable assumption that might explain this difference could be that the economic disparity between population subgroups is still minimal in Sri Lanka. Universal free access to health care in Sri Lanka could be another contributing factor.

The largest proportions of the sample were students and people with non-economic activities, such as housewives: however there was no significant difference between the employed and unemployed in HRQoL. In Sri Lanka, people with a higher education had better health than the non-educated, and this is consistent with the situation in high income countries (2,10). This indicates the disadvantaged suffer more from a lack of HRQoL than the advantaged even in a Low and Middle Income country like Sri Lanka. We tested the hypothesis that people with higher religiosity tend to be healthier: The results were mixed. Moderately religious people tend to have the highest utility score. However, the odds of reporting problems in any dimensions were highest for the deeply religious. A reasonable explanation could be moderately religious people may take comfort from their religion without getting too intense. It could also be due to reverse causality where people become more religious when they face with illness.

The present study used Sri Lankan EQ-5D-3L derived utility weights to calculate the health status of the sample. This is a strength of the study, and this could be argued to be a more valid approach than using another country's utility weights to calculate HRQoL population norms [15]. The present study found significant differences between the self-reported EQ-5D-3L VAS values and values produced from utility weights for the participants' EQ-5D-3L health states. However, many other population norms had been reported using EQ-5D-3L VAS values [6]. Future research is needed to find out whether EQ-5D-3L derived population norms should be confined to utility weights. However, this is the only population norm data published so far in the South Asian region and these can be used, as of now, in policy decisions. From this experience it is recommended to include the EQ-5D-3L questionnaire in the national housing and population census and demographic and health survey [30]. If the government of Sri Lanka can undertake such data collection the experience gained in the present analysis can be used to estimate better HRQoL information for Sri Lanka in the future.

There are some limitations to the present study. We covered only four districts of the country and the sample was skewed towards stay-at-home females. This was due to the time of the interview. The interview was conducted during the day and generally the households were occupied by females as males were away for work. We had a higher Sinhalese proportion in the sample as interviews were conducted either in Sinhalese or English. Logistic constrains prevented us from employing tri lingual data collectors. However, we had nearly 8% of Muslims in the sample. The Australian travel advice, at the time of the data collection, prevented us from collecting data from the predominantly Tamil, North and East of the country. However, as explained in the data analysis we did not weight the sample as population norms are presented by demographic groups. This places a caveat on the generalizability of the results to the Sri Lankan population as a whole. Considering the interaction between variables, it could be that younger people, who are less religious and are healthier than older people, drives the relationship between religiosity and HRQoL. It could be the tendency for older people to stay more at rural areas that make lesser HRQoL in the rural district.

## Conclusion

The use of the EQ-5D-3L to assess the health status of a population in a LMIC is feasible and informative. Population norms could be used for assessing effect of an intervention in non-randomised trials by allowing comparing with an index value. Using this instrument, an index value for “general” health status of population sub-groups can be estimated and used in the evaluation of population health. The health status of Sri Lankans shows decrement with age. Socioeconomically disadvantaged subgroups have lower health status than the more advantaged. Being a female was not a disadvantage in Sri Lanka. The trends observed in high income countries were generally similar to the Sri Lankan observations of population norms. Even though use of these population norms in decision making could prove challenging, the authors strongly urge their introduction.

## Supporting Information

Data Set S1 (XLSX)
